# Accuracy of routinely-collected healthcare data for identifying motor neurone disease cases: A systematic review

**DOI:** 10.1371/journal.pone.0172639

**Published:** 2017-02-28

**Authors:** Sophie Horrocks, Tim Wilkinson, Christian Schnier, Amanda Ly, Rebecca Woodfield, Kristiina Rannikmäe, Terence J. Quinn, Cathie L. M. Sudlow

**Affiliations:** 1 Centre for Clinical Brain Sciences, University of Edinburgh, Edinburgh, United Kingdom; 2 Institute of Cardiovascular and Medical Sciences, University of Glasgow, Glasgow, United Kingdom; 3 UK Biobank, Stockport, United Kingdom; Institute of Health Science, CHINA

## Abstract

**Background:**

Motor neurone disease (MND) is a rare neurodegenerative condition, with poorly understood aetiology. Large, population-based, prospective cohorts will enable powerful studies of the determinants of MND, provided identification of disease cases is sufficiently accurate. Follow-up in many such studies relies on linkage to routinely-collected health datasets. We systematically evaluated the accuracy of such datasets in identifying MND cases.

**Methods:**

We performed an electronic search of MEDLINE, EMBASE, Cochrane Library and Web of Science for studies published between 01/01/1990-16/11/2015 that compared MND cases identified in routinely-collected, coded datasets to a reference standard. We recorded study characteristics and two key measures of diagnostic accuracy—positive predictive value (PPV) and sensitivity. We conducted descriptive analyses and quality assessments of included studies.

**Results:**

Thirteen eligible studies provided 13 estimates of PPV and five estimates of sensitivity. Twelve studies assessed hospital and/or death certificate-derived datasets; one evaluated a primary care dataset. All studies were from high income countries (UK, Europe, USA, Hong Kong). Study methods varied widely, but quality was generally good. PPV estimates ranged from 55–92% and sensitivities from 75–93%. The single (UK-based) study of primary care data reported a PPV of 85%.

**Conclusions:**

Diagnostic accuracy of routinely-collected health datasets is likely to be sufficient for identifying cases of MND in large-scale prospective epidemiological studies in high income country settings. Primary care datasets, particularly from countries with a widely-accessible national healthcare system, are potentially valuable data sources warranting further investigation.

## Introduction

Motor neurone disease (MND) is a rare, rapidly progressive, neurodegenerative disease, which leads to muscle wasting, weakness and usually death within a few years of onset[1]. The aetiology is unclear and at present no cure is available. Further research that extends our current understanding of the aetiology and pathophysiology of the disease is urgently needed to bring us closer to developing effective treatment strategies.

Very large, population-based, prospective studies involving bio-sampling, detailed phenotyping and genotyping are ideal for investigating the determinants of diseases of complex aetiology, including neurodegenerative diseases such as MND. Through identifying sufficiently large numbers of incident cases of disease, such studies can provide adequate statistical power to detect associations of environmental, lifestyle, biological and genetic exposures with disease outcomes. They can also overcome the inherent limitations of retrospective case-control studies, including recall and reverse causation biases. A prominent example of such a study is UK Biobank, which recruited 500,000 participants aged 40–69 years old between 2006 and 2010, and has obtained a wealth of baseline information, stored bio-samples for current and future assays, additional post-recruitment phenotyping, genome-wide genotyping and consent for long term follow-up. Follow up of the participants’ health is chiefly via linkage to routinely-collected national health datasets such as hospital admissions, death registrations and primary care data. Data from UK Biobank are of substantial relevance to the international research community, since they are available to any bona fide researcher worldwide who wishes to conduct health-related research for the benefit of the public’s health[2].

Cohort-wide linkage to routinely-collected health datasets, especially within the context of a universally-available healthcare system such as the UK’s National Health Service (NHS), is a comprehensive and cost-efficient method of case identification for large prospective studies such as UKB. For aetiological research, the identification of disease cases within these cohorts must be of sufficient accuracy, with a high positive predictive value (PPV) and reasonable sensitivity. The accuracy of MND coding in these routinely-collected datasets therefore needs to be understood.

PPV refers to the proportion of cases identified by codes in routinely-collected health datasets that are true cases. Sensitivity refers to the proportion of true cases in a population that are identified by using codes in these health datasets. Specificity and negative predictive value (NPV) are less important accuracy measures for case-control comparisons nested within prospective studies as they tend to be high in these situations. In particular, NPV will be high when most individuals in the population do not have the disease in question.

In this study we aimed to systematically review all studies that investigated the accuracy of routinely-collected health datasets in identifying MND cases by comparing coded information to a reference standard.

In this paper we use the term ‘motor neurone disease’ as an umbrella term for the group of diseases of which amyotrophic lateral sclerosis (ALS) is one subtype, along with others such as progressive bulbar palsy and progressive muscular atrophy. Elsewhere, particularly in North America, the term ALS is used as the overarching term for this set of disorders. This difference in the use of the term ALS should be borne in mind when interpreting the results of studies in this review.

## Methods

### Study protocol

The protocol for this systematic review was published prospectively on PROSPERO (www.crd.york.ac.uk/PROSPERO, registration number 2015:CRD42015027985)[3].

### Search strategy

We searched MEDLINE (Ovid), EMBASE (Ovid), CENTRAL (Cochrane Library) and Web of Science (Thompson Reuters) for studies published between 1/1/1990-16/11/2015 that compared MND coding in routinely-collected datasets to a reference standard (see S1 Table for search criteria). We identified additional studies by searching the bibliographies of included studies and from personal communication. Two authors (SH and TW) independently screened all titles, abstracts and potentially relevant full text articles, resolving selection discrepancies through discussion and mutual consensus, and remaining areas of uncertainty through discussion with a third, senior author (CLMS).

### Eligibility criteria

Studies had to have been published in a peer-reviewed journal; to have compared routinely-collected, coded datasets using internationally recognised coding systems (e.g. International of Classification of Diseases, Read) to a reference standard for MND, based on medical diagnostic review; to have reported PPV, sensitivity or both (or provided data from which these could be calculated); and to have a sample size of ≥10 MND cases (since smaller studies would have limited precision). Studies estimating sensitivity had to have used a population-based reference standard, with comprehensive MND case ascertainment (e.g. a population-based MND register or similar). We did not impose any limitations based on published language or the country in which the study was conducted.

### Data extraction & analysis

Using pre-tested data extraction forms, two authors (SH and TW) independently extracted the following information: first author, publication year, country from which the relevant coded data were obtained, enrolment period, study population characteristics, study size, routine dataset(s) assessed (hospital, deaths, primary care), coding system, codes and coding positions (primary, secondary or any position) used to identify cases, reference standard used, PPV and/or sensitivity with their 95% confidence intervals (or data to calculate these). Any discrepancies were discussed and resolved with a third, senior author (CLMS). If data required to extract a PPV or sensitivity estimate were unclear or omitted from the published manuscripts, we contacted the original study authors for clarification.

Where appropriate, our approach used features of the methodology developed for systematic reviews of diagnostic test accuracy studies. However, there were key differences. In particular, for many studies that investigated PPV, it was not possible to also calculate sensitivity because the total number of MND cases (true positives and false negatives) in the relevant population was not known. We adapted the Quality Assessment of Diagnostic Accuracy Studies 2 (QUADAS-2) tool to evaluate study quality (S1 File)[4]. Two authors (SH & TW) completed the assessments of risk of bias and applicability (relevance to the study question) for the following QUADAS-2 categories: patient selection, source of coded data (including the codes used to identify cases), reference standard and study flow (e.g. whether all cases were accounted for). We assessed the risk of bias and the applicability of studies with respect to our review purpose, not on the quality of the paper in general. We did not exclude studies on the basis of quality.

Where data were available, we calculated 95% confidence intervals for PPV and sensitivity directly. We generated statistical measures of heterogeneity using I^2^ and chi-squared methods, but we focussed on descriptive assessments of heterogeneity based on evaluating study methodologies. We did not perform a formal meta-analysis as the substantial heterogeneity in methodologies between studies would make any summary measure of PPV or sensitivity potentially misleading. Instead, we performed a descriptive analysis, and considered factors that might influence PPV and sensitivity through visual inspection of the range of values in a forest plot. We also investigated within-study comparisons of PPV values with respect to characteristics reported by at least two studies (age, sex and coding position). We performed statistical analyses with StatsDirect3 software.

## Results

### Study selection

**Fig 1:**
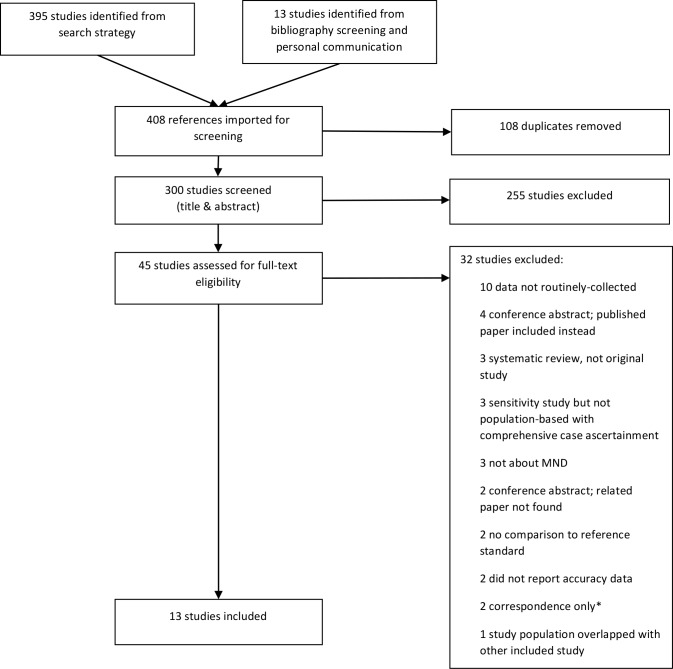
Selection of studies. *Correspondence: Short letters to journal editors and similar forms of communication

Thirteen studies fulfilled the eligibility criteria and were included in the review[5–17]. A flow diagram of the study selection process detailing reasons for exclusion is displayed in Fig 1.

### Study characteristics

Across the 13 studies there were 13 estimates of PPV[5–16] and five of sensitivity[7,8,16,17], with one study contributing separate PPV and sensitivity estimates for hospital and death data[7]. Characteristics of studies reporting PPV and sensitivity of a coded MND diagnosis are summarised in Tables 1 and 2 respectively. Comparison of the key study characteristics detailed in these tables reveals the heterogeneity of methodological approaches.

**Table 1:** Studies reporting the PPV of an MND code in routinely-collected datasets, separated by dataset evaluated.

Study	Country	Enrolment period	Study size (n)	Routine dataset	Coding system	Codes used to identify cases	Major coding position assessed	Disease investigated	Reference standard	Criteria for case confirmation	Notable study characteristics
Alonso 2009^5^	UK	1990–2005	65	P	Unclear	Unclear	Unclear	MND	Medical record review	Clinical judgement	Only cases with ≥1 year follow-up included
Beghi 2001^6^	Italy	1994–1995	237	H	ICD-9	335.2	Primary	ALS	Medical record review	El Escorial	Cases counted as TPs regardless of degree of El Escorial diagnostic certainty
Chancellor 1993^7^	UK	1989–1990	377	H	ICD-9	335	Any	MND	Register (Scottish Motor Neurone Disease Register (SMNDR))	Presence in the SMNDR	
Chió 2002^8^	Italy	1995–1996	433	H	ICD-9	335.2	Any	ALS	Register (The Piemonte and Valle d’Aosta Register for ALS (PARALS))	Presence in PARALS	
Doyle 2012^9^	UK	1996–2001	65	H	ICD-10	G12.2	Unclear	MND	GP confirmation	Medical record review	Women only
Drigo 2013^10^	Italy	2002–2009	332	H	ICD-9-CM	335.20	Any	ALS	Medical record review	Explicit diagnosis or riluzole prescription	
Fong 2005^11^	Hong Kong	1997–2002	217	H+O	ICD-9	335.xx	Unclear	MND	Patient examination or medical record review	Revised El Escorial	Unclear what degree of El Escorial diagnostic certainty counted as TPs
Kioumourtzoglou 2015^12^	Denmark	1982–2009	173	H	ICD-8; ICD-10	348 G12.2	Unclear	ALS	Medical record review	El Escorial	El Escorial possible cases counted as TP
Pisa 2009^13^	Italy	2005–2006	48	H	ICD-9-CM	335.2	Any	ALS	Medical record review	El Escorial	El Escorial suspected cases counted as TP s
Stickler 2011^14^	USA	2001–2005	336	H	ICD-9-CM	335.2	Any	ALS	Medical record review	Explicit documented diagnosis	
Chancellor 1993^7^	UK	1989–1990	281	D	ICD-9	335	Any	MND	Register (Scottish Motor Neurone Disease Register (SMNDR))	Presence in the SMNDR	
Stickler 2012^15^	USA	2001–2006	318	D	ICD-10	G12.2	Any	ALS	Medical record review	Clinical judgement	
Yeo 2010^16^	Republic of Ireland	2002–2006	397	D	ICD-9	Unclear	Any	MND	Register (Irish Register for ALS/MND)	Presence in Irish Register for ALS/MND	

Study size: Number of people with MND codes. Routine dataset: The routinely collected source of coded data. P: primary care data, H: hospital admissions data, H+O: hospital admissions and outpatient data, D: death certificate data. MND codes: ICD-8:348 = MND, 348.0 = amyotrophic lateral sclerosis, 348.1 = progressive bulbar palsy, 348.2 = other progressive muscular atrophy, 348.9 = Other and unspecified manifestations. ICD-9 & ICD-9-CM: 335.2 = MND, 335.20 = amyotrophic lateral sclerosis, 335.21 = progressive muscular atrophy, 335.22 = progressive bulbar palsy, 335.23 = pseudobulbar palsy, 335.24 = primary lateral sclerosis, 335.29 = other motor neurone disease. ICD-10: G12.2 = MND, G12.20 = unspecified, G12.21 = amyotrophic lateral sclerosis, G12.22 = progressive bulbar palsy, G12.29 = other motor neurone disease. Note: with respect to MND coding, ICD-9-CM (a clinical modification of ICD-9, used in North America) is identical to ICD-9. Coding position: Position at which a code for MND was assessed in the major analysis in the study. TP: true positive

**Table 2:** Studies reporting the sensitivity of an MND code in routinely collected datasets, separated by dataset evaluated.

First author	Country	Study period	Study population used to generate reference standard	Study size (n)	Routine dataset	Coding system	Code(s) assessed	Coding position assessed	Disease investigated	Sensitivity summary	Notable study characteristics
Chancellor 1993^7^	UK	1989–1990	MND cases as listed on the Scottish Motor Neurone Disease Register (SMNDR)	317	H	ICD-9	335	Any	MND	Proportion of MND cases with ICD-9 discharge code 335.2	
Chió 2002^8^	Italy	1995–1996	ALS cases as listed on The Piemonte and Valle d’Aosta Register for ALS (PARALS))	213	H	ICD-9	335.2	Any	ALS	Proportion of ALS cases with ICD-9 discharge code 335.2	Analysis limited to incident cases
Chancellor 1993^7^	UK	1989–1990	Deceased Scottish Motor Neurone Disease Register (SMNDR) cases.	95	D	ICD-9	335	Any	MND	Proportion of death certificates of known MND cases that report MND	
Chió 1992^17^	Italy	1970–1985	Deceased ALS cases ascertained from multiple overlapping sources (hospital archives, neurophysiology laboratories, social security records and files of neurologists)	488	D	Unclear	Unclear	Primary	ALS	Proportion of death certificates of known ALS cases that report ALS	
Yeo 2010^16^	Republic of Ireland	2002–2006	Deceased cases registered with the Irish Register for ALS/MND	398	D	ICD-9	Unclear	Any	ALS/MND	Proportion of death certificates of known ALS/MND cases that report ALS/MND	

Study population: Source of cases including method of case ascertainment. Study size: Number of known MND cases for which a code was sought. Routine dataset: The routinely collected source of coded datasets; H: hospital admissions data, D: death certification. MND codes: ICD-9 & ICD-9-CM: 335.2 = MND, 335.20 = amyotrophic lateral sclerosis, 335.21 = progressive muscular atrophy, 335.22 = progressive bulbar palsy, 335.23 = pseudobulbar palsy, 335.24 = primary lateral sclerosis, 335.29 = other motor neurone disease. ICD-10: G12.2 = MND, G12.20 = unspecified, G12.21 = amyotrophic lateral sclerosis, G12.22 = progressive bulbar palsy, G12.29 = other motor neurone disease. Coding position: Position at which a code for MND was assessed in the major analysis in the study.

All studies were based in high income countries. Three were from the UK[5,7,9], seven from other European countries[6,8,10,12,13,16,17], two from the USA[14,15] and one from Hong Kong [11]. For studies reporting PPV, sample size (number of participants with an MND code) ranged from 48–433; for those reporting sensitivity, sample size (number of participants known to have MND in the population-based reference standard) ranged from 95–488. The studies were conducted over a range of different time periods. Eight began before 2000, and three of these began prior to 1990. The vast majority of studies included assessment of hospital and/or death certificate-derived datasets, with only one study assessing primary care data[5].

Hospital and death data were coded using various different versions of the World Health Organisation International Classification of Diseases (ICD) system[18] (see Table 3). Based on the codes chosen, studies variably investigated all-cause MND, amyotrophic lateral sclerosis (ALS) or other MND subtypes. The single study that used primary care data did not report which coding system it used[5], but, since the study was UK-based, this is likely to have been the Read coding system, used since 1985 in UK primary care[19].

**Table 3:** International Classification of Diseases (ICD) codes for motor neurone disease and its subtypes.

ICD system	Code	Diagnosis
ICD-8	348	Motor neurone disease
	348.0	Amyotrophic lateral sclerosis
	348.1	Progressive bulbar palsy
	348.2	Other progressive muscular atrophy
	348.9	Other and unspecified manifestations
ICD-9	335.2	Motor neurone disease
	335.20	Amyotrophic lateral sclerosis
	335.21	Progressive muscular atrophy
	335.22	Progressive bulbar palsy
	335.23	Pseudobulbar palsy
	335.24	Primary lateral sclerosis
	335.29	Other motor neurone disease
ICD-10	G12.2	Motor neurone disease
	G12.20	Unspecified
	G12.21	Amyotrophic lateral sclerosis
	G12.22	Progressive bulbar palsy
	G12.29	Other motor neurone disease

The broad categories of diagnostic reference standard used were medical record review, presence in an MND patient register and direct patient assessment, although methodological details and diagnostic criteria for case confirmation varied. Four studies[6,11–13] used either the original or revised El Escorial criteria[20,21] to confirm a diagnosis of MND. These criteria require evidence of upper and lower motor neurone involvement, with a progressive spread of the regions affected. Depending on the clinical evidence obtained, the original El Escorial criteria identify five levels of diagnostic certainty: suspected, possible, laboratory-supported probable, probable and definite[20], while the revised El Escorial criteria identify three levels: possible, probable and definite[21]. Studies differed in the diagnostic certainty threshold required (Table 1).

### Quality assessment

Studies generally performed well in the subjective quality assessment (S2 Table). We did not consider any to be of high risk of bias or to have substantial applicability concerns. However, we rated 12 of the 13 studies as ‘unclear’ for at least one category, either because there was insufficient information to assess the category, or because we could not be sure what effect the reported methodology for that category would have on bias or applicability.

### PPV of routinely-collected datasets in MND case identification

**Fig 2:**
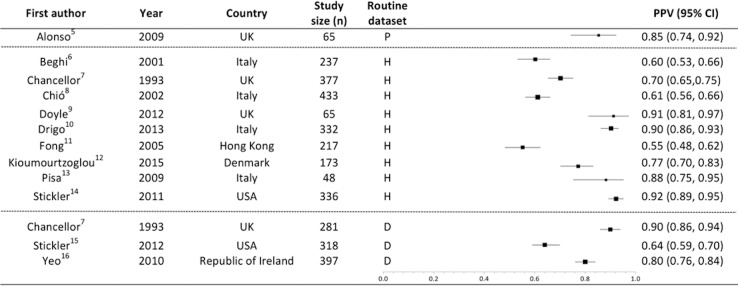
PPV of an MND code, stratified by type of routinely-collected dataset. Boxes weighted by study size. PPV: Positive predictive value. Study size: Number of cases with MND codes that were assessed. Routine dataset: The routinely collected source of coded datasets P: primary care data, H: hospital data, D: death certificate data. Heterogeneity measures: I^2^ = 97%, Chi-squared = 321.6 (df = 12) p<0.0001.

There were thirteen estimates of PPV, eight based on hospital admissions data[6–10,12–14], one on hospital admissions and outpatient data combined[11], three on death data[7,15,16] and one on primary care data[5]. PPV results stratified by data source are summarised in Fig 2. Reported PPVs ranged from 55%-92%; more than half of these (including over half of the total number of patients studied) were ≥80%. One included study that assessed hospital admissions data was of lower overall quality[12], but its PPV was not an outlier. PPVs for MND codes recorded in three studies of death certificate data ranged from 64–90%[7,15,16]; two of these (including around two thirds of the patients in the relevant studies) were ≥80%[7,16]. In the nine studies of hospital data, PPVs ranged from 55–92%[6–14]. Four of these nine studies reported PPVs >80%[9,10,13,14]. Based on the results of a single UK-based study, primary care data appeared to have a good PPV (85%)[5].

### Sensitivity of routinely-collected datasets in MND case identification

**Fig 3:**
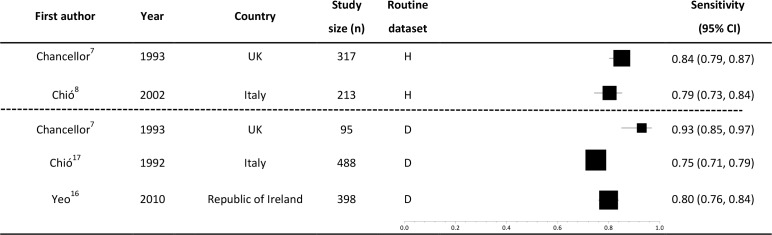
Sensitivity of an MND code, stratified by type of routinely-collected dataset. Boxes weighted by study size. Study size: Number of known MND cases for which a code was sought. Routine dataset: The routinely collected source of coded datasets. P: primary care data, H: hospital data, D: death certificate data. Heterogeneity measures: I^2^ = 83%, Chi-squared = 23.0 (df = 4) p = 0.0001.

There were five estimates of sensitivity, two from hospital discharge data[7,8] and three from mortality data[7,16,17] (Fig 3). No studies assessed the sensitivity of primary care data. All of the sensitivities reported were ≥75% and the values were less variable (range: 75–93%) than the PPVs. All studies reporting sensitivity were of high quality in the QUADAS-2 assessment. There was no observable difference between the sensitivity measures arising from death or hospital data. The studies assessing death data reported sensitivities of 75–93%[7,16,17] and those evaluating hospital data reported sensitivities of 79–84%[7,8].

### UK-based routinely-collected datasets

The UK NHS provides an ideal substrate for data linkage studies as there is a single provider of healthcare services. Of particular relevance for researchers worldwide using UK Biobank (and other large population-based UK cohorts that have linkage to routinely-collected healthcare datasets), the three UK-based studies reported some of the best performing results with respect to PPV and sensitivity[5,7,9] (Figs 2 and 3) and also scored well on the QUADAS-2 quality assessments.

### Within-study comparisons

Four studies conducted within-study analyses of the effects of age, gender or coding position on PPV estimates. However, sufficient data were not available to permit a consistent assessment of the statistical significance of the differences reported. Results are displayed in Table 4.

**Table 4:** Within-study sub-group analyses.

**The effect of age on PPV**					
**First author**	**Publication year**	**Routine dataset**	**Study size**	**Age**	**PPV**
Alonso^5^	2009	P	65	≤70	85%
				>70	85%
Chió^8^	2002	H	433	20–29	50%
				30–39	53%
				40–49	52%
				50–59	66%
				60–69	70%
				70–79	61%
				80–89	26%
Kioumourtzoglou^12^	2015	H	173	<55	78%
				55–74	82%
				>75	68%
**The effect of gender on PPV**					
**First author**	**Publication year**	**Routine dataset**	**Study size**	**Gender**	**PPV**
Chió^8^	2002	H	433	Male	60%
				Female	62%
Kioumourtzoglou^12^	2015	H	173	Male	78%
				Female	89%
**The effect of coding position on PPV**					
**First author**	**Publication year**	**Routine dataset**	**Study size**	**Coding position**	**PPV**
Chió^8^	2002	H	433	Any	61%
			309	Primary	74%
Stickler^14^	2011	H	336	Any	92%
			101	Primary	97%

PPV: Positive predictive value. Routine dataset: The routinely collected source of coded datasets. P: primary care data; H: hospital discharge data; D: death certification. Study size: Number of MND codes assessed (sum of true positives and false positives). TP: True positive. Number of TP cases: Total number of true positive cases, i.e. those for whom a diagnosis of MND was confirmed following application of reference standard. Coding position: Position at which a code for MND was recorded.

Two studies of hospital data and one of primary care data assessed the effect of age [5,8,12]. While the small primary care study found no difference in PPV between participants aged ≤70 and >70 years [5], the two larger studies of hospital data each reported a decline in PPV above the age of 70 to 75 years[8,12]. One of these reported that PPV increased with advancing age until ultimately falling in the elderly[8].

Two studies of hospital data examined the effect of sex on PPV[8,12]. Both reported a higher PPV in women. The difference was more substantial in one study (male PPV: 78%, female PPV: 89%)[12], than the other (male PPV: 60%, female PPV: 62%)[8].

Two studies of hospital data investigated the effect of the coding position of the recorded MND diagnosis [8,14]. Both found that codes in the primary position had a higher PPV than those in any position, but relying on the primary position alone substantially reduced the number of cases identified.

## Discussion

There is no widely-agreed level of the accuracy required for identifying disease cases for research using routinely-collected health datasets, and acceptable PPV and sensitivity thresholds will differ depending on the specific study purpose. In this systematic review we have shown that although reported accuracy estimates for identifying MND cases from such datasets vary widely, individual datasets often achieve PPV or sensitivity values of ≥80%, and can reach >90%.

False positive cases identified from coded data can be due to diagnostic or administrative errors. Given that–at least in many high income countries–the diagnosis of MND is usually made or confirmed by a specialist[22] we would expect diagnostic error to be low. However, clinical experience suggests that there are many patients in whom the diagnosis of MND is highly likely despite not meeting formal diagnostic criteria. Considering such patients to be ‘false positives’ in validation studies of coded data may result in falsely-low PPV estimates. The sensitivity of coded hospital admissions data for the identification of MND cases will depend on how likely MND patients are to be admitted to hospital during the course of their illness. This is likely to vary by geographic location, with differences in healthcare access and provision. Since MND usually leads to death within a few years of diagnosis, one would expect coded death data to be a sensitive source of MND case identification, as we observed.

Primary care data appears to be a promising source of MND case ascertainment for prospective studies based in countries with universally-accessible primary health care. Primary care in the UK is a free, comprehensive and lifelong service, in which general practitioners (GPs) act as gatekeepers to more specialist services, meaning that most individuals with an active diagnosis are likely to present to primary care at least once. Furthermore, GPs hold comprehensive medical notes for their patients, including correspondence from secondary care, resulting in diagnoses made in secondary care being coded in primary care datasets. Primary care data may therefore prove to be a rich resource for the study of MND epidemiology, particularly in countries without a national MND register. However, since only one small study reported the PPV of MND codes recorded in primary care data[5], and none reported the sensitivity, future investigation of the value of coded primary care data in MND case ascertainment is warranted.

Within-study analyses minimise confounding by variation in study methodology and setting, and so should enable more reliable evaluation of factors affecting the accuracy of case identification than between-study comparisons. However, such analyses were only available for a small number of studies and factors potentially influencing accuracy. While they showed that limiting case ascertainment to those recorded at the primary coding position may increase PPV, this was at the expense of the number of cases identified. In population-based, prospective studies such as UKB, methods of identifying disease cases with a high PPV are generally prioritised over those with a high sensitivity, as the effect of any false negatives (cases that are misidentified as controls) in case-control and case-cohort studies is diluted amongst the very large number of control subjects[23]. However, sensitivity needs to be sufficient to generate large numbers of cases for adequate statistical power as well as to ensure that representative cases are ascertained across the disease spectrum. It is important to strike a balance between the comprehensiveness of case ascertainment (reflected by high sensitivity) and the proportion of the pool of cases identified that are true positives (PPV).

### Heterogeneity of accuracy estimates

The wide range of reported PPV and sensitivity measures likely reflects variation in study methodologies as well as between the data sources.

The method of case confirmation (reference standard) could influence reported estimations of accuracy. Studies differed in their application of the El Escorial criteria, while subjects that could not be traced were counted as false positives in some studies but excluded from the analysis in others.

The system used to assign codes to diagnoses could also account for some variation. Most included studies assessed data coded using ICD-9 or ICD-10, which differ with respect to coding of MND subtypes: ICD-10 lists only ALS and progressive bulbar palsy as specific subtypes of MND, whereas ICD-9 permits sub-classification of five subtypes. However, variable study methods and characteristics precluded a reliable assessment of the effect of coding system on accuracy. A further issue relates to a problem with MND subgroup coding in an early version of ICD-10[9], in which the condition progressive supranuclear palsy (PSP) was wrongly given the code G12.2 for MND. This may affect the results of studies coded before this problem was rectified, as patients with PSP would have been given an MND code and then counted as false positives (e.g., Doyle et al. [2012] discovered that 8% of cases with the ICD-10 code G12.2 were miscoded due to this error[9]).

Variation may also arise from the specific codes chosen. Included studies variably investigated MND, ALS, and/or other specific disease sub-types. Studies that used a broad code, such as the ICD-9 335.2, would include rarer subtypes such as progressive muscular atrophy or primary lateral sclerosis (335.21 and 335.24 respectively) in addition to ALS, although the effect of including these very rare subtypes is likely to be minimal, as they are much less common than the ALS variant. More importantly, the choice of code to identify the relevant condition was sometimes inaccurate, leading to misclassification. For example, the ICD-9 code 335.2 which represents a diagnosis of MND, was often used interchangeably with code 335.20 representing ALS. Such usage may have led to the inappropriate classification of some outcomes as false positives within studies, but as clinical information for every possible case was not available, we were unable to determine the effect on results.

Although we cannot estimate their effects quantitatively, these methodological issues are likely to have caused spuriously low as opposed to falsely elevated PPV estimates, suggesting that the results generally represent minimum estimates of PPV.

### Increasing the accuracy of case identification

Accurate case ascertainment may be optimised by an algorithm which draws upon multiple sources, to improve both PPV and sensitivity. For example, one study that did not meet the eligibility criteria for this review as it combined routine and non-routine data sources (insurance data, death registrations, reports from local neurologists and records from the ALS Association) achieved an improvement in PPV from 84% with single sources to 98% with combined sources[24]. An additional method of improving PPV might be to only include cases that appear more than once within a dataset or in more than one dataset. Where possible, linkage to robust, comprehensive, national disease registers such as the population-based Scottish MND Register[25] is likely to be a powerful way to increase both sensitivity and PPV.

### Strengths and limitations

Our review benefits from rigorous methodology, including prospective protocol publication, comprehensive search criteria, and involvement of two independent authors in study screening, quality assessments and data extraction. While some relevant studies may have been missed, our extensive search criteria should minimise this possibility. We included all identified, eligible studies to retain a comprehensive, systematic approach and avoid study selection bias. While including studies of lower quality could theoretically affect our results, such studies did not have extreme PPV or sensitivity values. Publication or selective reporting biases could have influenced our results, since studies showing high accuracy might be published or reported more often than those with lower accuracy. However, such effects are difficult to assess meaningfully in this type of review, and so we did not attempt formally to estimate these potential biases. Lastly, PPV increases with the prevalence of the condition of interest in the study population, meaning that PPVs will tend to be higher for common conditions. We were unable to assess the underlying prevalence of MND across the study populations, but given that MND is generally rare, we believe this is unlikely to have substantially affected variability of our PPV estimates.

## Conclusions

In general, PPV and sensitivity of routinely-collected health data in identifying MND cases are likely to be sufficient for many epidemiological studies investigating the determinants of MND. However, in view of the range of reported results, prospective studies may wish to perform their own validation studies to evaluate the PPV and/or sensitivity for their particular study setting and population. For UK Biobank, which has obtained primary care data for many participants, further studies that assess the improvements in accuracy achieved by identifying MND cases through primary care data in addition to hospital and death data, will be helpful. In the meantime, scientists interested in using UK Biobank or other UK-based prospective studies with data linkage for MND-related research can be reassured that PPV and sensitivity in UK studies of hospital admissions and death registration data are among the highest reported for MND, while the one UK-based primary care study showed promising results. In view of the different advantages associated with each type of dataset and the additional factors that influence the accuracy of a coded diagnosis of MND, the development of a case identification algorithm based on multiple overlapping sources may be particularly valuable and merits further investigation.

## Supporting information

S1 Checklist PRISMA checklist. (PDF)

S1 Table Search Strategy. (PDF)

S2 Table QUADAS-2 results. ✔ Low Risk**?** Unclear Risk ✘ High Risk.
(PDF)

S1 File Adapted QUADAS-2 form. (PDF)

S2 File Members of the UK Biobank Follow-up and Outcomes Working Group and Neurodegenerative Outcomes Advisory Group. (PDF)

## References

[1] Worms PM. The epidemiology of motor neuron diseases: a review of recent studies. J Neurol Sci. 2001;191: 3–9. 11676986 10.1016/s0022-510x(01)00630-x

[2] Sudlow C, Gallacher J, Allen N, Beral V, Burton P, Danesh J, et al UK biobank: an open access resource for identifying the causes of a wide range of complex diseases of middle and old age. PLoS Med. 2015;12: e1001779 10.1371/journal.pmed.1001779 25826379 PMC4380465

[3] Horrocks S. The accuracy of routinely collected health datasets in identifying motor neuron disease cases in population-based prospective studies: a systematic review. PROSPERO 2015:CRD42015027985. Available: www.crd.york.ac.uk/PROSPERO/display_record.asp?ID=CRD42015027985.

[4] Whiting PF, Rutjes AWS, Westwood ME, Mallett S, Deeks JJ, Reitsma JB, et al QUADAS-2: a revised tool for the quality assessment of diagnostic accuracy studies. Ann Intern Med. 2011;155: 529–536. 10.7326/0003-4819-155-8-201110180-00009 22007046

[5] Alonso A, Logroscino G, Jick SS, Hernán MA. Incidence and lifetime risk of motor neuron disease in the United Kingdom: a population-based study. Eur J Neurol. 2009;16: 745–751. 19475756 10.1111/j.1468-1331.2009.02586.x PMC3093130

[6] Beghi E, Logroscino G, Micheli A, Millul A, Perini M, Riva R, et al Validity of hospital discharge diagnoses for the assessment of the prevalence and incidence of amyotrophic lateral sclerosis. Amyotroph Lateral Scler Mot Neuron Disord. 2001 6;2(2):99–104. 10.1080/146608201316949541 11675878

[7] Chancellor AM, Swingler RJ, Fraser H, Clarke JA, Warlow CP. Utility of Scottish morbidity and mortality data for epidemiological studies of motor neuron disease. J Epidemiol Community Health. 1993;47: 116–120. 8326268 10.1136/jech.47.2.116 PMC1059738

[8] Chiò A, Ciccone G, Calvo A, Vercellino M, Di Vito N, Ghiglione P, et al Validity of hospital morbidity records for amyotrophic lateral sclerosis. A population-based study. J Clin Epidemiol. 2002;55: 723–727. 12160921 10.1016/s0895-4356(02)00409-2

[9] Doyle P, Brown A, Beral V, Reeves G, Green J. Incidence of and risk factors for motor neurone disease in UK women: a prospective study. BMC Neurol. 2012;12: 25 10.1186/1471-2377-12-25 22559076 PMC3512483

[10] Drigo D, Verriello L, Clagnan E, Eleopra R, Pizzolato G, Bratina A, et al The incidence of amyotrophic lateral sclerosis in Friuli Venezia Giulia, Italy, from 2002 to 2009: a retrospective population-based study. Neuroepidemiology. 2013;41: 54–61. 10.1159/000350015 23711404

[11] Fong GCY, Cheng TS, Lam K, Cheng WK, Mok KY, Cheung CM, et al An epidemiological study of motor neuron disease in Hong Kong. Amyotroph Lateral Scler Mot Neuron Disord. 2005 9;6(3):164–8. 10.1080/14660820510028412a 16247937

[12] Kioumourtzoglou M-A, Seals RM, Himmerslev L, Gredal O, Hansen J, Weisskopf MG. Comparison of diagnoses of amyotrophic lateral sclerosis by use of death certificates and hospital discharge data in the Danish population. Amyotroph Lateral Scler Front Degener. 2015;16: 224–229. 10.3109/21678421.2014.988161 PMC4675622 25946516

[13] Pisa FE, Verriello L, Deroma L, Drigo D, Bergonzi P, Gigli GL, et al The accuracy of discharge diagnosis coding for Amyotrophic Lateral Sclerosis in a large teaching hospital. Eur J Epidemiol. 2009;24: 635–640. 10.1007/s10654-009-9376-1 19657715

[14] Stickler DE, Royer JA, Hardin JW. Validity of hospital discharge data for identifying cases of amyotrophic lateral sclerosis. Muscle Nerve. 2011;44: 814–816. 10.1002/mus.22195 22006696

[15] Stickler DE, Royer JA, Hardin JW. Accuracy and usefulness of ICD-10 death certificate coding for the identification of patients with ALS: results from the South Carolina ALS Surveillance Pilot Project. Amyotroph Lateral Scler. 2012 1;13(1):69–73. 10.3109/17482968.2011.614253 21929354

[16] Yeo L, Lynch C, Hardiman O. Validating population-based registers for ALS: how accurate is death certification? J Neurol. 2010;257: 1235–1239. 10.1007/s00415-010-5494-7 20151145

[17] Chiò A, Magnani C, Oddenino E, Tolardo G, Schiffer D. Accuracy of death certificate diagnosis of amyotrophic lateral sclerosis. J Epidemiol Community Health. 1992;46: 517–518. 1479322 10.1136/jech.46.5.517 PMC1059643

[18] World Health Organization. ICD-10 Version:2016. 2016. Available: http://apps.who.int/classifications/icd10/browse/2016/en

[19] NHS Digital. Read Codes. 2016. Available: http://systems.digital.nhs.uk/data/uktc/readcodes

[20] Brooks BR. El Escorial World Federation of Neurology criteria for the diagnosis of amyotrophic lateral sclerosis. Subcommittee on Motor Neuron Diseases/Amyotrophic Lateral Sclerosis of the World Federation of Neurology Research Group on Neuromuscular Diseases and the El Escorial “Clinical limits of amyotrophic lateral sclerosis” workshop contributors. J Neurol Sci. 1994;124 Suppl: 96–107. 7807156 10.1016/0022-510x(94)90191-0

[21] Brooks BR, Miller RG, Swash M, Munsat TL, World Federation of Neurology Research Group on Motor Neuron Diseases. El Escorial revisited: revised criteria for the diagnosis of amyotrophic lateral sclerosis. Amyotroph Lateral Scler Mot Neuron Disord. 2000 12;1(5):293–9. 10.1080/146608200300079536 11464847

[22] NICE. Motor neurone disease: assessment and management. 2016. Available: https://www.nice.org.uk/guidance/ng42

[23] Woodfield R, Grant I, Sudlow CLM. Accuracy of Electronic Health Record Data for Identifying Stroke Cases in Large-Scale Epidemiological Studies: A Systematic Review from the UK Biobank Stroke Outcomes Group. PLoS ONE. 2015;10(10):e0140533 10.1371/journal.pone.0140533 26496350 PMC4619732

[24] Benatar M, Wuu J, Usher S, Ward K. Preparing for a U.S. National ALS Registry: Lessons from a pilot project in the State of Georgia. Amyotroph Lateral Scler. 2011 3;12(2):130–5. 10.3109/17482968.2010.515224 20843169

[25] The Scottish Motor Neuron Disease Research Group. The Scottish Motor Neuron Disease Register: a prospective study of adult onset motor neuron disease in Scotland. Methodology, demography and clinical features of incident cases in 1989. J Neurol Neurosurg Psychiatry. 1992;55: 536–541. 1640227 10.1136/jnnp.55.7.536 PMC489161

