# Supplementary material for: Accuracy of routinely-collected healthcare data for identifying motor neurone disease cases: A systematic review
Source: PLoS One. 2017 Feb 28;12(2):e0172639. doi: 10.1371/journal.pone.0172639 (PMC5330471; doi:10.1371/journal.pone.0172639)
Supplement: S1 Table — (PDF) [file pone.0172639.s002.pdf]

S1 Table. Search Strategy

Search criteria were developed with the help of an information specialist.

MEDLINE search strategy (Ovid MEDLINE(R) In-Process & Other Non-Indexed Citations and Ovid MEDLINE(R) 1946 to Present)

1	"International Classification of Diseases"/ or "international classification of diseas*".mp. or "ICD ten*".mp. or ICD10.mp. or "ICD 10".mp. or "ICD 9".mp. or ICD9.mp. or "ICD nine".mp. or ICD-9-CM.mp. or ICD-10-CM.mp. or "administrat* data".mp. or "medical record*".mp. or "health information*".mp. or claim*.mp. or "hospital discharge*".mp. or "inpatient discharge*".mp. or "hospital episode*".mp. or "hospital episode statistics".mp. or "scottish morbidity record*".mp. or SMR*.mp. or "patient episode database for wales".mp. or PEDW.mp. or coding.mp. or code*.mp. or exp Clinical Coding/ or "medical record review".mp. or exp Information Systems/ or exp Medical Records/ or exp medical records systems, computerized/ or exp electronic health records/ or exp Electronic Health Records/exp or Primary Health Care/exp or general practice/ or exp family practice/ or "read cod*".mp. or exp Patient Discharge/ or exp Patient Discharge Summaries/ or exp Hospital Records/ or exp Health Services Research/ or "physician claims".mp. or "death certificate*".mp. or exp death certificates/ or exp hospital records/ or "death registration*".mp. or case ascertainment.mp. or medicare or exp health insurance/ or exp Outpatients/ or "outpatient data".mp.
2	(sensitivity or specificity).mp. or exp "sensitivity and specificity"/ or ((pre-test or pretest) adj probability).mp. or exp "Predictive Value of Tests"/ or "predictive value*".mp. or "likelihood ratio*".mp. or exp validation studies/ or "validation stud*".mp. or "positive predictive value".mp. or exp "reproducibility of results"/ or "reproducibility of results".mp. or "positive predictive value".mp. or "negative predictive value".mp. or validity.mp. or reproducibility.mp. or accuracy.mp. or agreement.mp. or validation.mp. or algorithm*.mp. or exp algorithms/ or (identif* adj3 dement*).ti,ab. or (detect* adj3 dement*).ti,ab. or (ROC or "receiver operat*").ab. or sROC.ab. or Area Under Curve/
3	"amyotrophic lateral sclerosis".mp. or exp Amyotrophic Lateral Sclerosis/ or "progressive bulbar palsy".mp. or exp Bulbar Palsy, Progressive/ or "primary lateral sclerosis".mp. or "progressive muscular atrophy".mp. or "motor neuron* disease".mp. or exp Motor Neuron Disease/ or "anterior horn cell disease".mp.
4	1 and 2 and 3
5	limit 4 to yr="1990 –Current”

EMBASE search strategy – (Embase 1980 to Present)

1	"International Classification of Diseases"/ or "international classification of diseas*".mp. or "ICD ten*".mp. or ICD10.mp. or "ICD 10".mp. or "ICD 9".mp. or ICD9.mp. or "ICD nine".mp. or ICD-9-CM.mp. or ICD-10-CM.mp. or "administrat* data".mp. or "medical record*".mp. or "health information*".mp. or claim*.mp. or "hospital discharge*".mp. or "inpatient discharge*".mp. or "hospital episode*".mp. or "hospital episode statistics".mp. or "scottish morbidity record*".mp. or SMR*.mp. or "patient episode database for wales".mp. or PEDW.mp. or coding.mp. or code*.mp. or exp Clinical Coding/ or "medical record review".mp. or exp Information Systems/ or exp Medical Records/ or exp medical records systems, computerized/ or exp electronic health records/ or exp Electronic Health Records/exp or Primary Health Care/exp or general practice/ or exp family practice/ or "read cod*".mp. or exp Patient Discharge/ or exp Patient Discharge Summaries/ or exp Hospital Records/ or exp Health Services Research/ or "physician claims".mp. or "death certificate*".mp. or exp death

	certificates/ or exp hospital records/ or "death registration*".mp. or case ascertainment.mp. or medicare or exp health insurance/ or exp Outpatients/ or "outpatient data".mp.
2	(sensitivity or specificity).mp. or exp "sensitivity and specificity"/ or ((pre-test or pretest) adj probability).mp. or exp "Predictive Value of Tests"/ or "predictive value*".mp. or "likelihood ratio*".mp. or exp validation studies/ or "validation stud*".mp. or "positive predictive value".mp. or exp "reproducibility of results"/ or "reproducibility of results".mp. or "positive predictive value".mp. or "negative predictive value".mp. or validity.mp. or reproducibility.mp. or accuracy.mp. or agreement.mp. or validation.mp. or algorithm*.mp. or exp algorithms/ or (identif* adj3 dement*).ti,ab. or (detect* adj3 dement*).ti,ab. or (ROC or "receiver operat*").ab. or sROC.ab. or Area Under Curve/
3	"amyotrophic lateral sclerosis".mp. or exp Amyotrophic Lateral Sclerosis/ or "progressive bulbar palsy".mp. or exp Bulbar Palsy, Progressive/ or "primary lateral sclerosis".mp. or "progressive muscular atrophy".mp. or "motor neuron* disease".mp. or exp Motor Neuron Disease/ or "anterior horn cell disease".mp.
4	1 and 2 and 3
5	limit 4 to yr="1990 –Current”

Cochrane Library search strategy – (Cochrane Reviews (Reviews only), Other Reviews, Trials, Methods Studies, Technology Assessments, Economic Evaluations and Cochrane Groups)

1	“international classification of diseases” OR “ICD*” OR “administrat* data” OR “medical record*” OR “health information” OR claim* OR “hospital discharge*” OR “inpatient discharge*” OR “hospital episode*” OR “hospital episode statistics” OR “scottish morbidity record” OR “patient episode database for wales” OR coding OR code* OR “medical record*” OR “electronic health record*” OR “hospital record*” OR medicare OR “health insurance”
2	“positive predictive value” OR “negative predictive value” OR accuracy OR sensitivity OR specificity OR validity
3	“amyotrophic lateral sclerosis” OR “Motor Neuron Disease” OR “Motor Neurone Disease”
4	1 and 2 and 3 (Year from 1990 to 2015)

Web of Science search strategy – (Web of Science Core Collection)

1	TS=(“international classification of diseases” OR “ICD*” OR “administrat* data” OR “medical record*” OR “health information” OR claim* OR “hospital discharge*” OR “inpatient discharge*” OR “hospital episode*” OR “hospital episode statistics” OR “scottish morbidity record” OR “patient episode database for wales” OR coding OR code* OR “medical record*” OR “electronic health record*” OR “hospital record*” OR medicare OR “health insurance”)
2	TS=(“positive predictive value” OR “negative predictive value” OR accuracy OR sensitivity OR specificity OR validity)
3	TS=(“amyotrophic lateral sclerosis” OR “Motor Neuron Disease” OR “Motor Neurone Disease”)
4	1 and 2 and 3 (*Timespan=1990-2015)*
