# Supplementary material for: Accuracy of routinely-collected healthcare data for identifying motor neurone disease cases: A systematic review
Source: PLoS One. 2017 Feb 28;12(2):e0172639. doi: 10.1371/journal.pone.0172639 (PMC5330471; doi:10.1371/journal.pone.0172639)
Supplement: S2 Table — ✔ Low Risk? Unclear Risk ✘ High Risk. (PDF) [file pone.0172639.s003.pdf]

S2 Table. QUADAS-2 results

First author	Year	Risk of bias: Patient selection	Risk of bias: Index test	Risk of bias: Reference standard	Risk of bias: Flow and timing	Applicability concerns: Patient selection	Applicability concerns: Index test	Applicability concerns: Reference standard
Alonso	2009	✓	?	✓	✓	✓	✓	✓
Beghi	2001	✓	✓	?	?	✓	✓	?
Chancellor	1993	✓	✓	?	?	✓	✓	✓
Chió	1992	?	✓	✓	✓	✓	?	✓
Chió	2002	✓	✓	✓	?	✓	✓	✓
Doyle	2012	?	✓	✓	✓	✓	✓	✓
Drigo	2013	✓	✓	?	✓	?	✓	✓
Fong	2005	?	✓	✓	?	✓	?	✓
Kioumourtzoglou	2015	?	✓	?	?	?	?	✓
Pisa	2009	?	✓	✓	✓	✓	✓	✓
Stickler	2011	✓	✓	✓	✓	✓	✓	✓
Stickler	2012	✓	✓	✓	?	✓	✓	✓
Yeo	2010	✓	?	?	✓	✓	✓	✓
