# Supplementary material for: Accuracy of routinely-collected healthcare data for identifying motor neurone disease cases: A systematic review
Source: PLoS One. 2017 Feb 28;12(2):e0172639. doi: 10.1371/journal.pone.0172639 (PMC5330471; doi:10.1371/journal.pone.0172639)
Supplement: S1 File — (PDF) [file pone.0172639.s004.pdf]

S1 File. Adapted QUADAS-2 form

QUADAS-2 assessment: The accuracy of electronic health datasets in identifying motor neuron disease cases in population-based prospective studies: a systematic review

Primary study:

Flowchart:

1. **Patient selection**

a) Risk of bias: *Could the selection of patients have introduced bias?*
Describe methods of patient selection: *Consider whether the criteria used to identify MND cases will pick up a consecutive or random sample or simply all cases within a specific subpopulation e.g. a certain geographical region. Does the study only include patients presenting in a certain manner, or with a specific reason for contact with the health system (e.g. PEG/NIV etc.) or patients with a given severity of disease?*

Was a consecutive or random sample of patients enrolled?	Yes / No / Unclear
Did the study avoid inappropriate exclusions?<br>*Does the study avoid excluding patients whose principal diagnosis/ presenting complaint was not related to MND, but who had MND as a co-morbidity? (This would not be appropriate if the study is investigating the accuracy of the datasets in determining prevalence, but may be appropriate if incidence is being studied).*	Yes / No / Unclear
Could the selection of patients have introduced bias?<br>Risk: Low/ High/ Unclear	

b) Applicability assessment: *Are there concerns that the included patients and setting do not match the review question?*
Describe methods of patient selection and included patients. *Consider if patients with other conditions (e.g. other forms of anterior horn cell disease picked up from ICD-9 335 coding) or if healthy individuals may be identified and/or included in the group of cases. Does the setting of the study and the demographic features of the study cohort reflect the wider population? Is the study population large enough? Does the method of patient selection aim to pick up incident or prevalent cases?*
Is there concern that the included patients do not match the review question?<br>Concern: Low / High / Unclear

2. Index test

a) Risk of bias: *Could the conduct or interpretation of the index test have introduced bias?*	
Describe the index test and how it was conducted and interpreted: *Consider if the index test may introduce bias e.g. are presentations to primary care likely to be less severe/complex than hospital presentations and what effect will this have on making the diagnosis? If MND is more frequently diagnosed in hospital (inpatient or outpatient setting) than in primary care this may influence the accuracy of diagnosis. For what reason(s) have the individuals been recorded in the administrative dataset? Does the study avoid only including presentations to healthcare that are likely to occur in earlier or later stages of the disease? Does the study only include first hospital admissions/presentations, or does it also include repeat admissions/presentations? If hospital data was investigated does the study describe the features of the hospital, as they may have some influence on the diagnostic accuracy; size of hospital, location, teaching hospital, presence of neuro dept.; public/private hospital clinical training of the person making the diagnosis or performing the diagnostic coding etc. Were only certain wards included and does hospital data include only discharge data, or only outpatient data, or possibly both? Similar variables to those outlined above will need to be considered and described for data from general practice and possibly also from death registrations.*	
Were the index test results interpreted without knowledge of the results of the reference standard?	Yes / No / Unclear
If a threshold was used, was it pre-specified?<br>*Consider if the study includes possible/probable cases of MND*	Yes / No / Unclear
Did the study avoid inappropriate exclusions?<br>*See above. Consider what influences presentation to GP/ hospital and mortality data, as MND patients who do not present to medical/healthcare will be excluded.*	Yes / No / Unclear
Could the conduct or interpretation of the index test have introduced bias?<br>Risk: Low / High / Unclear	

b) Applicability assessment: *Are there concerns that the index test, its conduct, or interpretation differ from the review question?*
See above. Does the index test conform to the definition of a routinely collected dataset?<br><br>Concern: Low / High / Unclear

3. Reference standard

a) <u>Risk of bias</u>: *Could the reference standard, its conduct, or its interpretation have introduced bias?*	
Describe the reference standard and how it was conducted and interpreted: *Alternatives include retrospective evaluation of medical notes by an expert clinician, clinical assessment of the patient, which may involve the application of diagnostic criteria such as El Escorial or Awaji as well as disease registers such as SMNDR (Scottish Motor Neuron Disease Register). Is the gold standard classification performed by somebody with sufficient knowledge and experience to make this diagnosis accurately? Similarly, a sufficient amount of high quality medical documentation is necessary for retrospective review of clinical documentation. Was the clinician who performed the assessment blinded to the results of the index test? If an 'expert clinician' performed the evaluation, what level of expertise do they have? If >1 clinician/researcher do they have the same level of expertise?*	
Is the reference standard likely to correctly classify the target condition?	Yes / No / Unclear
Were the reference standard results interpreted without knowledge of the results of the index test?	Yes / No / Unclear
Could the reference standard, its conduct, or its interpretation have introduced bias?<br>Risk: Low / High / Unclear	

b) <u>Applicability assessment</u>: Are there concerns that the target condition as defined by the reference standard does not match the question?
Describe the reference standard and how it was conducted and interpreted: *See above. Is the gold standard used to classify the degree of diagnostic certainty into suspected, possible, probable and definite ALS and if so which groups are included in statistical analysis? For example including cases of uncertain diagnosis may lead to overestimation of sensitivity. If a disease register is used, is it well established and has it been shown to have ~100% case ascertainment? For the first few years following establishment of a register case identification may still be low. What diagnostic criteria was used e.g. AWAJI criteria, El Escorial criteria or the preceding simplified WFN diagnostic criteria? Consider the relative merits of each.*<br><br>Concern: Low / High / Unclear

4. Flow and timing

a) Risk of bias: Could the patient flow have introduced bias?	
Describe any patients who did not receive the index test(s) and/or reference standard or who were excluded from the 2x2 table (refer to flow diagram): *Can all patients be followed through the study? Are any patients not included in analysis that were originally identified? If so, why? For example exclusion of patients based on residency may not be appropriate. If data from general practice or hospital discharges used, excluding patients who die before the end of the study may not be appropriate.*	
Describe the time interval and any interventions between index test(s) and reference standard:	
Was there an appropriate interval between index test and reference standard? *For example are the reference cases obtained from the same time period as that over which the health datasets are assessed? Using populations from different time periods and assuming that the incidence and prevalence are the same would be a flaw.*	Yes / No / Unclear
Did all patients receive a reference standard?	Yes / No / Unclear
Did patients receive the same reference standard? *Did the same 'expert' perform the assessment for reference standard? If diagnostic criteria were used were the same diagnostic criteria used throughout? Was there verification by a second 'expert' clinician in any/all cases?*	Yes / No / Unclear
Were all patients included in the analysis?	Yes / No / Unclear
Could the patient flow have introduced bias?<br>Risk: Low / High / Unclear	
