# Supplementary material for: Accuracy of routinely-collected healthcare data for identifying motor neurone disease cases: A systematic review
Source: PLoS One. 2017 Feb 28;12(2):e0172639. doi: 10.1371/journal.pone.0172639 (PMC5330471; doi:10.1371/journal.pone.0172639)
Supplement: S2 File — (PDF) [file pone.0172639.s005.pdf]

S2 File. Members of the UK Biobank Follow-up and Outcomes Working Group and Neurodegenerative Outcomes Advisory Group

UK Biobank Follow-up and Outcomes Working Group
John Danesh, Cambridge University
Naomi Allen, UK Biobank, Oxford University
Mark Atkinson, Swansea University
Ekaterini Blaveri, Cancer Research UK
Rachael Brannan, National Cancer Intelligence Network
Carol Brayne, Cambridge University
Sinead Brophy, Swansea University
Nish Chaturvedi, University College London
Rory Collins, UK Biobank, Oxford University
Simon deLusignan, Surrey University
Spiros Denaxas, University College London
Parul Desai, Moorfields Eye Hospital
Sophie Eastwood, University College London
John Gallacher, Cardiff University
Harry Hemingway, University College London
Matthew Hotopf, Kings College London
Martin Landray, Oxford University
Ronan Lyons, Swansea University
Mark McGilchrist, Dundee University
Henrik Moller, Kings College London
Terence O'Neil, Manchester University
Mike Pringle, Nottingham University
Tim Sprosen, Oxford University
David Strachan, St George's University, London
Cathie Sudlow, UK Biobank, Edinburgh University
Frank Sullivan, Dundee University
Rebecca Woodfield, Edinburgh University
Qiuli Zhang, UK Biobank, Edinburgh University
Robin Flaig, UK Biobank, Edinburgh University

UK Biobank Neurodegenerative Outcomes Working Group
Naomi Allen, UK Biobank, Oxford University
Roger Barker, Cambridge University
Yoav Ben-Shlomo, University of Bristol
Carol Brayne, Cambridge University
Andrew Bucknor, Oxford University
Camille Carroll, Plymouth University
Siddharthan Chandran, University of Edinburgh
Carl Counsell, University of Aberdeen
John Danesh, Cambridge University
Daniel Davis, University of Edinburgh
Katrina Davis, King's College London
Mike Denis, Oxford University
Mick Dennis, Swansea University
Pat Doyle, London School of Hygiene and Tropical Medicine

Nick Fox, University College London
John Gallacher, Cardiff University
Valentina Gallo, Queen Mary University of London
Jane Green, Oxford University
Matthew Hotopf, King’s College London
Simon Lovestone, Oxfrod University
Ronan Lyons, Swansea University
Ian McKeith, Newcastle University
Craig Newman, Plymouth University
John O’Brian, Cambridge University
Suvankar Pal, University of Edinburgh
Sarah Pendlebury, Oxford University
Craig Ritchie, University of Edinburgh
Martin Rossor, University College London
John Starr, University of Edinburgh
Blossom Stephan, Newcastle University
Rob Stewart, King’s College London
Cathie Sudlow, UK Biobank, University of Edinburgh
Robert Swingler, University of Edinburgh
Will Whiteley, University of Edinburgh
Tim Wilkinson, University of Edinburgh
